# Lateral Collateral Ligament Calcification: A Rare and Challenging Cause of Chronic Knee Pain

**DOI:** 10.7759/cureus.98799

**Published:** 2025-12-09

**Authors:** José Eduardo Sousa, Nuno Madureira, Paula Freire, Maria João Sousa, Carolina Paiva

**Affiliations:** 1 Physical Medicine and Rehabilitation, Centro de Medicina de Reabilitação da Região Centro - Rovisco Pais, Coimbra, PRT

**Keywords:** calcific tendinopathy, conservative treatment, knee pain, lateral collateral ligament, magnetic resonance imaging, shockwave therapy, ultrasound imaging

## Abstract

Lateral collateral ligament (LCL) calcification of the knee represents an uncommon degenerative or post-traumatic change characterized by ectopic mineral deposition within the ligamentous fibers. We report a rare case of LCL calcification in a 49-year-old female presenting with chronic right lateral knee pain with recurrent acute exacerbations. The patient had a prior history of mild knee trauma, and no significant findings were initially identified on radiographs. Despite several courses of physiotherapy and oral nonsteroidal anti-inflammatory drugs (NSAIDs), her symptoms persisted with only partial relief. Physical examination revealed localized tenderness over the lateral knee compartment and pain with varus stress testing. Bedside ultrasound revealed a well-circumscribed hyperechoic lesion within the LCL consistent with calcific deposition. MRI confirmed the diagnosis. A conservative management plan consisting of radial extracorporeal shockwave therapy (rESWT) was initiated, leading to complete symptom resolution at three-month follow-up.

## Introduction

Calcific deposition within periarticular soft tissues is a well-known phenomenon, most commonly affecting tendons such as those of the rotator cuff or gluteal region [1,2]. However, calcification of the lateral collateral ligament (LCL) of the knee is exceedingly rare, with only a few cases documented in the literature [1-4]. This entity is characterized by hydroxyapatite crystal deposition within the ligamentous fibers, resulting in localized inflammation, pain, and sometimes restricted motion [1,3]. Its precise etiology remains uncertain, though factors such as previous trauma, repetitive microtrauma, or metabolic disturbances may play a role [1,2].

Radiologically, LCL calcification can mimic more aggressive or traumatic lesions, including avulsion fractures, myositis ossificans, or even neoplastic processes [1,4]. Differentiating it from other causes of lateral knee pain - such as ligament sprain, meniscal tear, or iliotibial band syndrome - is crucial for its proper management [1,2]. Most cases respond favorably to conservative measures, including nonsteroidal anti-inflammatory drugs (NSAIDs), physiotherapy, and ultrasound-guided interventions [3,5]. We report a chronic case successfully treated with a noninvasive approach, highlighting the diagnostic value of ultrasound and MRI in managing this rare condition [3].

## Case presentation

A 49-year-old female with no significant past medical history presented to our Physical Medicine and Rehabilitation (PMR) clinic with a two-year history of persistent right lateral knee pain. The pain had a mechanical pattern, exacerbated by prolonged standing or walking for more than 15 minutes, and was occasionally accompanied by localized swelling and stiffness. The patient recalled a motorcycle accident approximately six years earlier, with no reported fractures or ligamentous injury. Over the years, she had undergone multiple cycles of physiotherapy and several courses of NSAIDs, which provided only temporary relief.

**Figure 1:**
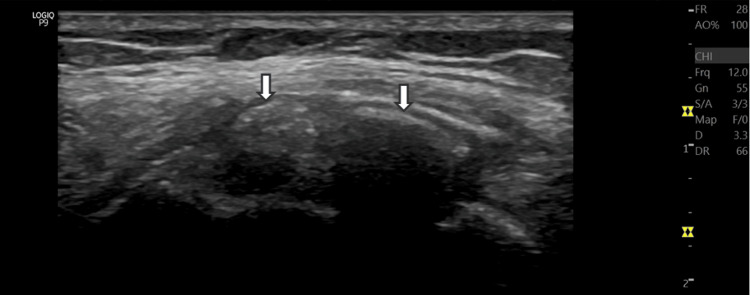
Ultrasound image Longitudinal ultrasound image showing focal calcification foci (arrows) within the proximal fibers of the LCL LCL: lateral collateral ligament

**Figure 2:**
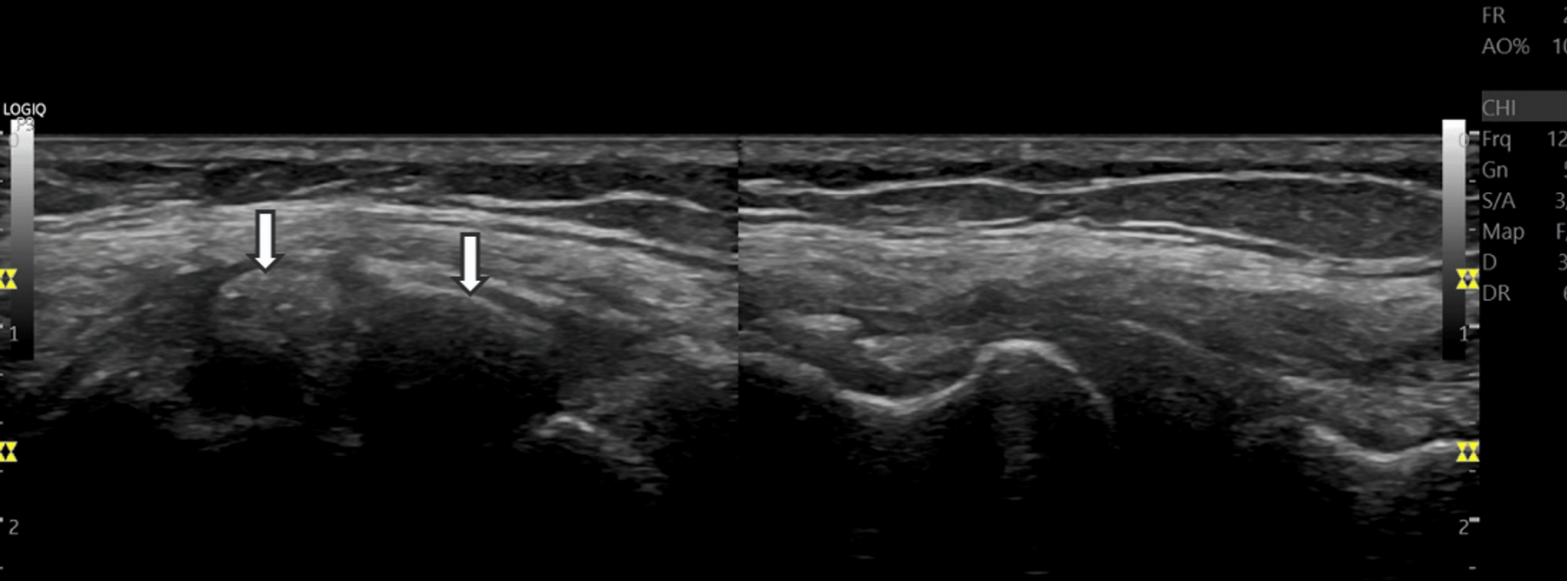
Comparative ultrasound image Comparative assessment with asymptomatic left knee (right half of the image), where LCL demonstrates a normal fibrillar and echoic pattern, with intact fibers and no evidence of calcification or thickening LCL: lateral collateral ligament

Physical examination revealed pain during varus stress testing, without joint instability or effusion, and point tenderness over the lateral aspect of the right knee. Range of motion was preserved, and no abnormalities were observed in gait or patellar tracking. Ultrasound evaluation demonstrated two hyperechoic foci within the proximal third of the LCL, measuring approximately 2.5 and 5 mm in diameter, with posterior acoustic shadowing, consistent with a calcific deposit. The adjacent bone surface appeared intact, and no joint effusion or evidence of ligament disruption was present (Figures 1, 2).

**Figure 3:**
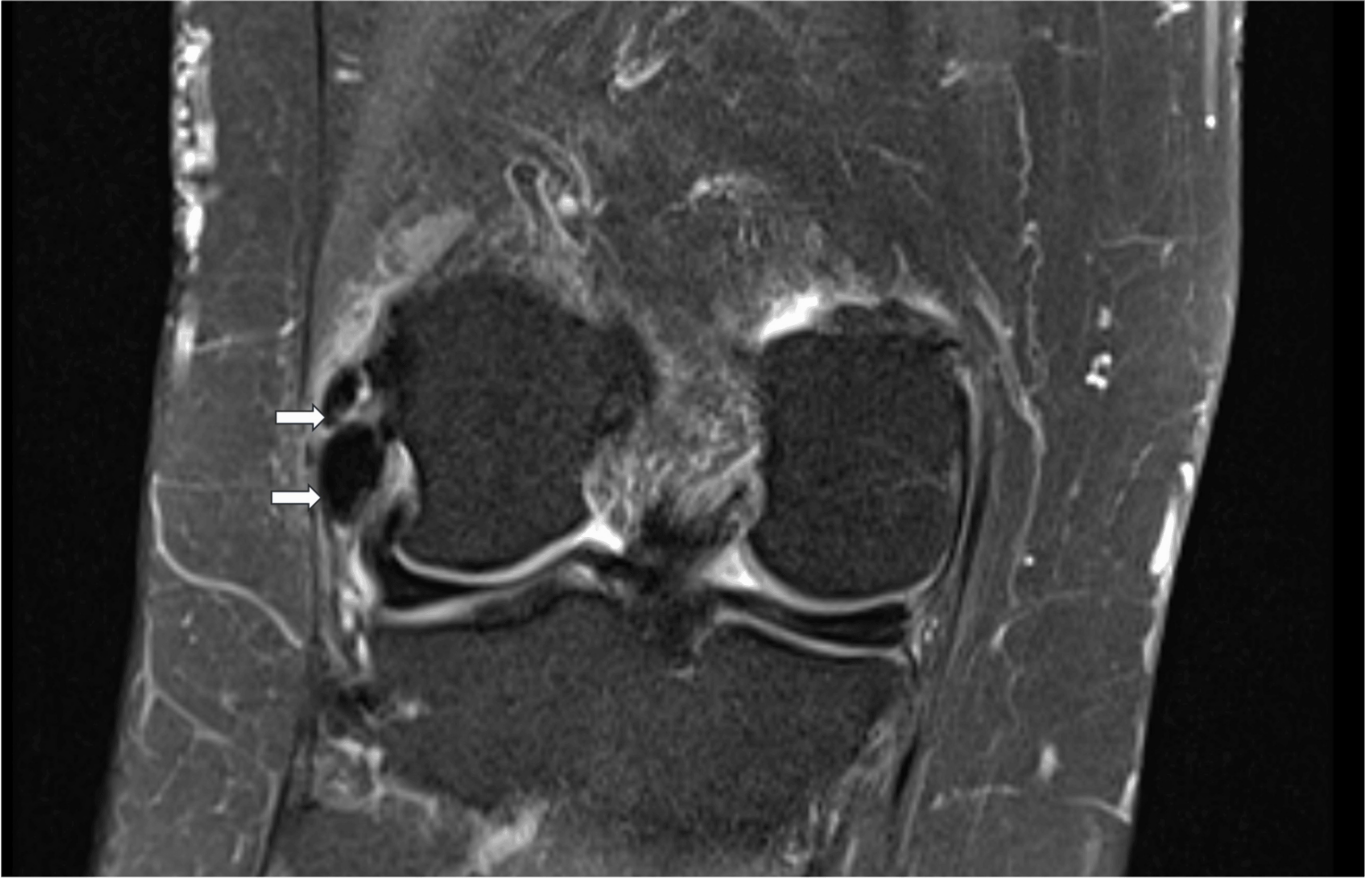
MRI image Coronal T2-weighted MRI image showing calcifications in the LCL (arrows) MRI: magnetic resonance imaging; LCL: lateral collateral ligament

Laboratory results were unremarkable (Table 1). For diagnostic clarification and further evaluation, an MRI was obtained, which demonstrated two small, well-defined low-signal-intensity foci within the proximal fibers of the LCL on both T1- and T2-weighted sequences, consistent with calcification. There was no evidence of ligament discontinuity, surrounding bone marrow edema, or soft-tissue mass. The menisci and cruciate ligaments were intact, and there was no joint effusion or chondral defect (Figure 3). Overall, the findings were compatible with calcific ligamenthopathy of the LCL without associated structural damage.

**Table 1:** Laboratory test results Test results were within normal ranges, including markers to assess calcium metabolism and calcification risk

Variable	Result	Units	Reference range
Hemoglobin (Hb)	13.8	g/dL	13.0 – 17.0
Hematocrit (HCT)	41.5	%	40 – 50
Red blood cells (RBC)	4.7	x10⁶/µL	4.5 – 5.9
Mean corpuscular volume (MCV)	88	fL	80 – 96
Mean corpuscular Hb concentration (MCHC)	33	g/dL	32 – 36
White blood cells (WBC)	6.8	x10³/µL	4.0 – 10.0
Neutrophils	58	%	40 – 75
Lymphocytes	32	%	20 – 45
Monocytes	6	%	2 – 10
Eosinophils	3	%	0 – 6
Basophils	1	%	0 – 2
Platelets	245	x10³/µL	150 – 400
C-reactive protein (CRP)	0.4	mg/dL	<0.5
Lactate dehydrogenase (LDH)	180	U/L	140 – 280
Alkaline phosphatase (ALP)	84	U/L	44 – 147
Gamma-glutamyl transferase (GGT)	22	U/L	8 – 61
Creatinine	0.9	mg/dL	0.6 – 1.2
Magnesium	2.0	mg/dL	1.7 – 2.4
Total serum calcium	10.3	mg/dL	8.5 – 10.5
Ionized calcium	1.31	mmol/L	1.12 – 1.32
Phosphate (phosphorus)	3.2	mg/dL	2.5 – 4.5
Albumin	4.1	g/dL	3.5 – 5.0
Parathyroid hormone (PTH)	48	pg/mL	10 – 65
25(OH) vitamin D	32	ng/mL	30 – 100

Given the chronic nature of symptoms and poor response to prior conservative management, a regimen of radial extracorporeal shockwave therapy (rESWT) was initiated - comprising five sessions at weekly intervals, using 2000 impulses per session at 2.0 bar pressure and 10 Hz frequency. This was complemented by a tailored physiotherapy program emphasizing gentle stretching, proprioceptive training, and quadriceps/hamstring strengthening to optimize knee biomechanics and reduce lateral compartment overload. At the three-month follow-up, the patient reported complete resolution of pain and a return to full activity levels, including regular walking and cycling. No recurrence of symptoms was observed at six months.

## Discussion

LCL calcification is an uncommon cause of lateral knee pain and may often go unrecognized due to its nonspecific clinical presentation [1-4]. The pathophysiology is thought to parallel that of calcific tendinitis elsewhere, progressing through three stages: formative, resting, and resorptive [1,3]. During the resorptive phase, inflammatory mediators are released, leading to acute pain and swelling. Chronic presentations, such as in this case, may reflect a protracted resorptive phase or incomplete resolution [3]. It is currently understood that the LCL lies along the outer aspect of the knee and, in the absence of a nearby bursa, provides no localized environment for hydroxyapatite deposition, which explains the rarity of calcification in this ligament [1,2].

Imaging plays a central role in diagnosis. Plain radiographs may miss small, early, or late deposits [1,4]. Ultrasound provides real-time visualization of calcific foci and guides minimally invasive treatments such as barbotage or lavage [3]. MRI, while sensitive, may show low-signal foci that mimic more aggressive pathologies, especially when associated with soft tissue edema [1,4]. Recognition of characteristic imaging features is therefore crucial to avoid unnecessary interventions or misdiagnosis [1-4]. The differential diagnosis for periarticular calcifications includes post-traumatic dystrophic calcification, calcium pyrophosphate deposition disease (CPPD), gouty tophus, endocrine/metabolic disorders (hyperparathyroidism, renal osteodystrophy), and infectious or neoplastic processes [1,2]. In our patient, the absence of systemic findings and normal laboratory parameters supported a presumptive diagnosis of localized, idiopathic hydroxyapatite deposition process [1,3].

The management of calcific musculoskeletal lesions is primarily conservative. First-line treatment includes relative rest, NSAIDs, and targeted physical therapy aimed at restoring function and correcting muscle-tendon imbalances [5]. In cases where pain persists despite these measures, extracorporeal shockwave therapy (ESWT) has shown encouraging results [5]. Radial waves, characterized by lower energy and greater superficial dispersion compared to focused waves, exert their therapeutic effects through both biomechanical and biological mechanisms that promote neovascularization, enhancement of local metabolism, and stimulation of tissue regeneration [5].

In the setting of calcific musculoskeletal pathologies, shockwaves appear to facilitate the fragmentation and resorption of calcium deposits within the tendon, promoting their clearance through macrophage-mediated cellular processes [5]. Furthermore, the mechanical stimulation induced by shockwaves is thought to modulate the expression of growth factors such as VEGF (vascular endothelial growth factor) and BMP-2 (bone morphogenetic protein-2), creating a microenvironment conducive to tendon repair [5]. Due to its more superficial and diffuse mode of action, rESWT is particularly suitable for superficial calcific deposits, offering a well-tolerated and effective noninvasive treatment option in the outpatient setting [5]. Our patient’s complete recovery following rESWT supports its efficacy and safety in managing chronic LCL calcification [5].

## Conclusions

LCL calcification is a rare but clinically relevant cause of chronic lateral knee pain that may mimic more serious conditions. Accurate diagnosis relies on a combination of clinical suspicion and multimodal imaging, particularly ultrasound and MRI. This report highlights the importance of considering calcific deposition as part of the differential diagnosis in chronic lateral knee pain. Conservative treatment combining physiotherapy and rESWT demonstrated efficacy, achieving symptom resolution and restoration of function while avoiding invasive procedures. Early recognition and appropriate management of this condition can help prevent surgical procedures and promote favorable long-term outcomes.
